# Systematic review and meta-analysis on the prognostic value of complete remission status at FDG-PET in Hodgkin lymphoma after completion of first-line therapy

**DOI:** 10.1007/s00277-015-2529-2

**Published:** 2015-10-20

**Authors:** Hugo J. A. Adams, Rutger A. J. Nievelstein, Thomas C. Kwee

**Affiliations:** Department of Radiology and Nuclear Medicine, University Medical Center Utrecht, Heidelberglaan 100, 3584 CX Utrecht, The Netherlands

**Keywords:** FDG-PET, Hodgkin lymphoma, Meta-analysis, Systematic review, Treatment

## Abstract

This study aimed to systematically review and meta-analyze the prognostic value of complete remission status at ^18^F-fluoro-2-deoxy-D-glucose positron emission tomography (FDG-PET) in Hodgkin lymphoma after completion of first-line therapy. A systematic literature search was performed in the MEDLINE database for suitable original articles. The included studies were methodologically assessed using the Quality In Prognosis Studies tool. The proportion of patients who developed disease relapse during follow-up, among those patients who were in complete remission according to FDG-PET at the completion of first-line therapy, was calculated for each included study. Heterogeneity in disease relapse proportions across individual studies was assessed using the *I*^2^ statistic, with heterogeneity regarded present if *I*^2^ < 50 %. Weighted summary disease relapse proportion was calculated using either a random effects model (if *I*^2^ > 50) or a fixed effects model (if *I*^2^ ≤ 50). Ten studies comprising a total number of 1137 Hodgkin lymphoma patients with complete remission status according to FDG-PET after completion of first-line therapy were included. Overall methodological quality of included studies was reasonably good. The disease relapse rate during follow-up among all patients with complete remission status at end-of-treatment FDG-PET ranged from 0 to 26.7 %, with a weighted summary proportion of 7.5 % (95 % confidence interval 3.9–13.8 %) using the random effects model (*I*^2^ = 88.3 %). In conclusion, although the disease relapse rate in Hodgkin lymphoma patients who achieve an FDG-PET-based complete remission after first-line therapy is low from an absolute point of view, it is actually high when considering the generally favorable outcome of Hodgkin lymphoma.

## Introduction

Hodgkin lymphoma is a relatively rare cancer, accounting for less than 1 % of newly diagnosed cancer cases with an annual incidence of two to three per 100,000 in the Western world [1, 2]. Early favorable Hodgkin lymphoma is defined by stage I/II disease without risk factors such as large mediastinal mass, extranodal disease, elevated erythrocyte sedimentation rate, or involvement of three or more nodal areas [3]. In contrast, patients with stage I/II disease presenting with one or more of these risk factors are usually allocated to the early unfavorable risk group, while patients with stage III/IV disease have advanced Hodgkin lymphoma [3]. At present, patients diagnosed with early-stage disease mostly receive combined-modality therapy consisting of two to four cycles of chemotherapy (usually with adriamycin, bleomycin, vinblastine, and dacarbazine [ABVD]) followed by involved-field radiation therapy [3]. Advanced Hodgkin lymphoma is usually treated with six to eight cycles of chemotherapy (either with ABVD or escalated bleomycin, etoposide, adriamycin, cyclophosphamide, vincristine, procarbazine, and prednisone [BEACOPP]), optionally followed by localized radiation therapy [3].

Although more than 80 % of all Hodgkin lymphoma patients can be cured with standard first-line therapy [4], early identification of patients who will relapse after standard therapy is important to start alternative effective treatments in a timely manner. High-dose chemotherapy followed by autologous stem cell transplantation has become the standard therapy for refractory or relapsed patients and leads to long-term cure in more than 50 % of patients [2]. Meanwhile, other promising drugs such as the antibody drug conjugate brentuximab vedotin are being investigated in these patients [2, 5]. There is an active search for new prognostic biomarkers in Hodgkin lymphoma [3]. At present, the International Prognostic Score (IPS) is the best validated clinical risk stratification index to predict the outcome of patients with newly diagnosed advanced Hodgkin lymphoma [3, 6, 7], while positron emission tomography (PET) with the glucose analogue ^18^F-fluoro-2-deoxy-D-glucose (FDG) is considered as the standard of care for remission assessment in Hodgkin lymphoma [8–10]. A meta-analysis on the prognostic value of end-of-treatment FDG-PET in Hodgkin lymphoma that was performed more than 8 years ago reported that FDG-PET seems to have a good diagnostic accuracy for assessing residual Hodgkin lymphoma at the completion of first-line treatment [11]. However, the 15 studies on Hodgkin lymphoma that were included in that meta-analysis had considerable heterogeneity and suboptimal methodologic quality and reporting [11]. Thus, although end-of-treatment FDG-PET is widely used in clinical practice to assess complete remission status in Hodgkin lymphoma, compelling evidence on its prognostic value is still lacking.

The purpose of this study was therefore to systematically review and meta-analyze the prognostic value of complete remission status at FDG-PET in Hodgkin lymphoma after completion of first-line therapy.

## Materials and methods

### Search strategy

The MEDLINE database was searched using the PubMed interface for original articles on the prognostic value of complete remission status at FDG-PET in Hodgkin lymphoma after completion of first-line therapy, from start date to 11 December 2014. The search strategy consisted of a combination of synonyms of the terms “FDG-PET” and “Hodgkin.” Bibliographies of included studies were checked for suitable references that were not retrieved by the initial MEDLINE search.

### Study selection

Original studies (either retrospective or prospective) investigating the prognostic value of complete remission status at FDG-PET in Hodgkin lymphoma after completion of first-line therapy (with or without additional radiation therapy) were eligible for inclusion. Initially, no language restriction was applied. Studies lacking original patient data such as review articles, management guidelines, editorials, and letters were excluded. Studies with less than 10 Hodgkin lymphoma patients, case reports, and conference abstracts were also excluded. Potentially eligible articles from the same authors or institution were evaluated for duplication of data. If this was the case, only the most recent article was included. Studies that included patients with other malignancies or lymphoma subtypes than Hodgkin lymphoma who could not be separated from Hodgkin lymphoma patients, studies that included patients with disease refractory to first-line therapy, studies that included patients who had received second-line therapy who could not be separated from patients who had received first-line therapy, studies that only included Hodgkin lymphoma patients who had a residual mass at end-of-treatment computed tomography (CT), studies in which complete remission status was not based on FDG-PET findings, studies in which the Cheson criteria [8–10] incorporating FDG-PET were not used, and studies from which the number of disease relapses among patients who were in complete remission according to end-of-treatment FDG-PET could not be extracted were excluded. At the first stage, titles and abstracts of all retrieved studies were screened and studies not meeting the above-mentioned inclusion criteria were excluded. At the second stage, the full-text versions of the remaining studies were obtained and evaluated using the above-mentioned inclusion and exclusion criteria.

### Study quality

The quality of included studies was critically appraised using the Quality In Prognosis Studies (QUIPS) tool, which was developed to assess the risk of bias in studies of prognostic factors [12]. The QUIPS tool assesses the risk of bias in six different domains: study participation (“Does the study population adequately represent the population of interest?”), study attrition (“Do the study data available [i.e., patients not lost to follow-up] adequately represent the study sample?”), prognostic factor measurement (“Is the prognostic factor measured in a similar way for all participants?”), outcome measurement (“Is the outcome of interest measured in a similar way for all participants?”), study confounding (“Have important potential confounding factors appropriately been accounted for?”), and statistical analysis and reporting (“Is the statistical analysis appropriate, and are all primary outcomes reported?”) [12]. Risk of bias in each of these six domains was scored as “low,” “moderate,” or “high” [12].

### Statistical analysis

The proportion of patients who developed disease relapse during follow-up, among those patients who were in complete remission according to FDG-PET at the completion of first-line therapy, was calculated for each included study. Heterogeneity in disease relapse proportions across individual studies was assessed using the *I*^2^ statistic, with heterogeneity regarded present if *I*^2^ > 50 % [13]. Weighted summary disease relapse proportion was calculated using either a random effects model (if there was heterogeneity across individual studies) or a fixed effects model (if there was no heterogeneity across individual studies). Note that patients with residual or progressive disease according to end-of-treatment FDG-PET were not included in this analysis, because these patients may undergo additional treatments that influence outcome. A similar separate analysis was done for patients who had undergone stand-alone FDG-PET instead of integrated FDG-PET/CT and for patients with either early stage or advanced stage disease, if these patient data could be extracted from the original studies. Comprehensive Meta-Analysis Version 2.2.064 (Biostat, Englewood, Illinois, USA) was used for statistical analysis.

## Results

### Literature search

The MEDLINE search yielded 1627 articles. After reviewing titles and abstracts, 139 potentially eligible articles remained. After reviewing the full-text versions of these articles, 56 were excluded because they did not allow extracting the number of disease relapses of patients who were in complete remission according to end-of-treatment FDG-PET, 19 were excluded because complete remission status was not based on FDG-PET findings in all patients, 18 were excluded because the Cheson criteria [8–10] were not used, 12 were excluded because they only included patients with a residual mass at end-of-treatment CT, 12 were excluded because the study did not allow separate data extraction of Hodgkin lymphoma patients from patients with other malignancies, nine were excluded because 10 or less patients with Hodgkin lymphoma were included, one was excluded because it reported data that were also used in another (larger) study, one was excluded because it included patients with refractory disease, and one study was excluded because it was written in Chinese. Thus, 10 studies [14–23] with a total of 1137 patients were finally included in this systematic review and meta-analysis. The characteristics of included studies and patients and the applied study methodology are displayed in Tables 1 and 2.

**Table 1:** Characteristics of included studies and patients

Study (year)	Country	Data acquisition	No. of patients^a^	Age in years (range)	Sex (M/F)	Stage (no.)	IPS score/EORTC risk factor group	Treatment regimes (no.)
Hutchings et al. (2014) [14]	Multinational	Prospective	126	34.1^c^ (16.8–76.7)	59/67	I, 10 IIA, 34 IIB, 24 III, 24 IV, 34	Mean 2.15 Range 0–6^e^	ABVD, 121 BEACOPPesc, 5 Additional RT, 44
Picardi et al. (2014) [15]	Italy	Prospective	300	29^c^ (18–70)	181/119	IIB, 55 IIIA, 75 IIIB, 85 IVA, 20 IVB, 65	0–1, 91 2–3, 121 4–7, 88^e^	6× ABVD, 300 Additional RT, 131
Filippi et al. (2013) [16]	Italy	Retrospective	80	29^c^ (15–72)	24/56	IA, 2 IIA, 78	Favorable, 33 Unfavorable, 47^f^	3× ABVD, 9 4× ABVD, 71
Markova et al. (2012) [17]	Czech Republic	Retrospective	69	30.7^d^	29/40	IIB, 13 IIIA, 5 IIIB, 21 IVA, 3 IVB, 14	0–1, 30 2–3, 28 4–7, 11^e^	8× BEACOPPesc, *n* = 35 6× BEACOPPesc, *n* = 24 8× BEACOPP_14_, 18
Okosun et al. (2012) [18]	UK	Retrospective	23^b^	42^c^ (32–60)	20/3	II, 7 III, 6 IV, 10	≥3, 14 <3, 9^e^	ABVD + HAART
Zinzani et al. (2012) [19]	Italy	Retrospective	304	32^c^ (13–78)	150/154	IA/B, 11 IIA, 136 IIB, 57 IIIA/B, 62 IVA/B, 38	NR	ABVD 6× (214) or ABVD 4× + RT (90)
Barnes et al. (2011) [20]	USA	Retrospective	96	34^c^ (18–77)	44/52	IA, 11 IB, 1 IIA, 63 IIB, 21	NR	4× ABVD, 1 6× ABVD, 41 4× ABVD + RT, 25 6× ABVD + RT, 29
Lopci et al. (2011) [21]	Italy	Retrospective	98	13.8^d^	NR	I, 10 II, 56 III, 12 IV, 20	NR	ABVD COPP + ABV ABVD + IEP ABVD + IEP + COPP
Straus et al. (2011) [22]	USA	Prospective	99	37^c^ (18–80 years)	49/50	IA, 10 IIA, 70 IIB, 19	Favorable, 11 Unfavorable, 69^f^	6× AVG
Cerci et al. (2010) [23]	Brazil	Prospective	104	28^c^ (13–82)	55/49	I, 2 II, 41 III, 25 IV, 36	0–2, 62 3–7, 42^e^	4–6× ABVD, 43 6–8× ABVD, 25 8× ABVD, 36 Additional RT

*ABVD* adriamycin, bleomycin, vinblastine, and dacarbazine, *AVG* adriamycin, vinblastine, and gemcitabine, *BEACOPP* bleomycin, etoposide, adriamycin, cyclophosphamide, vincristine, procarbazine, and prednisolone, *COPP* cyclophosphamide, oncovin, procarbazine, and prednisolone, *EORTC* European Organisation for Research and Treatment of Cancer, *IEP* ifosfamide, etoposide, and prednisolone, *IPS* International Prognostic Score, *NR* not reported, *RT* radiation therapy

^a^Of all patients who were included in this study (including those without complete remission status at end-of-treatment FDG-PET)

^b^All patients who were included in this study were HIV-positive

^c^Median

^d^Mean

^e^IPS score [6]

^f^EORTC risk factor group [33]

**Table 2:** FDG-PET imaging and interpretation methods, and criteria for disease relapse that were used in the included studies

Study (year)	Imaging system(s)	FDG dose	Time between FDG administration and scanning	Time between last therapy cycle and FDG-PET	Interpreters	Baseline scan available (No.)	Reference standard for disease relapse
Hutchings et al. (2014) [14]	Hybrid PET/CT	NR	60–90 min	NR	Two experienced readers	Yes	Confirmation by histology
Picardi et al. (2014) [15]	Hybrid PET/CT	5.3 MBq/kg	60 ± 10 min	NR	Nuclear medicine physicians and radiologist	NR	Confirmation by histology
Filippi et al. (2013) [16]	Hyrid PET/CT	NR	NR	3 months	NR	Yes	NR
Markova et al. (2012) [17]	Stand-alone PET	NR	NR	2–6 weeks	Central review panel	Yes, 58 No, 11	NR
Okosun et al. (2012) [18]	Hybrid PET/CT	NR	NR	NR	A nuclear medicine physician	Yes, 20 No, 3	NR
Zinzani et al. (2012) [19]	Stand-alone PET	5.3 MBq/kg	60–90 min	1–3 months	Two nuclear medicine physicians	Yes	Confirmation by histology
Barnes et al. (2011) [20]	Stand-alone PET or hybrid PET/CT	370–740 MBq	±60 min	NR	Two nuclear medicine physicians	NR	NR
Lopci et al. (2011) [21]	Stand-alone PET or hybrid PET/CT	NR	NR	NR	Nuclear medicine physicians	NR	Clinical monitoring, other imaging modalities, follow-up scans, or histology
Straus et al. (2011) [22]	NR	NR	NR	NR	Two independent reviewers and an adjudicator	Yes	Histology, 18 Follow-up, 5
Cerci et al. (2010) [23]	Stand-alone PET	296–444 MBq	60 min	NR	Two nuclear medicine physicians	Yes	Confirmation by histology

### Methodological quality assessment

The six QUIPS domains with risk of bias for each of the 10 included studies are displayed in Table 3. Overall, the methodological quality of included studies was reasonably good. Nevertheless, there was moderate risk of bias for the domain of prognostic factor measurement in six studies, because five studies [17, 19–21, 23] used a stand-alone PET system rather than integrated PET/CT, and one study did not report at all which PET system was used [22]. In addition, there was moderate risk of bias for the domain of outcome measurement in five studies, because three studies [16, 17, 20] did not report whether all disease relapses were confirmed by histology and two studies [21, 22] reported that histology was not used to confirm disease relapse in all cases.

**Table 3:** Quality assessment of included studies (risk of bias in six different domains according to the QUIPS tool [12])

Study (year)	Study participation	Study attrition	Prognostic factor measurement	Outcome measure	Study confounding	Statistical analysis
Hutchings et al. (2014) [14]	Low	Low	Low	Low	Low	Low
Picardi et al. (2014) [15]	Low	Low	Low	Low	Low	Low
Filippi et al. (2013) [16]	Low	Low	Low	Moderate	Low	Low
Markova et al. (2012) [17]	Low	Low	Moderate	Moderate	Low	Low
Okosun et al. (2012) [18]	Low	Low	Low	Low	Low	Low
Zinzani et al. (2012) [19]	Low	Low	Moderate	Low	Low	Low
Barnes et al. (2011) [20]	Low	Low	Moderate	Moderate	Low	Low
Lopci et al. (2011) [21]	Low	Low	Moderate	Moderate	Low	Low
Straus et al. (2011) [22]	Low	Low	Moderate	Moderate	Low	Low
Cerci et al. (2010) [23]	Low	Low	Moderate	Low	Low	Low

### Prognostic performance

All included studies provided data on the number of disease relapses that occurred during follow-up. The disease relapse rate during follow-up among all patients with complete remission status at FDG-PET after completion of first-line therapy ranged from 0 to 26.7 % (Table 4), with a weighted summary proportion of 7.5 % (95 % confidence interval 3.9–13.8 %) using the random effects model (*I*^2^ = 88.3 %). In patients who had undergone stand-alone FDG-PET instead of integrated FDG-PET/CT (three studies [17, 19, 23], the disease relapse rate during follow-up ranged from 5.1 to 11.8 %, with a weighted summary proportion of 7.0 % (95 % confidence interval 3.7–12.9 %) using the random effects model (*I*^2^ = 60.3 %). In patients with early stage disease, the disease relapse rate during follow-up ranged from 1.3 to 13.8 %, with a weighted summary proportion of 5.7 % (95 % confidence interval 2.5–12.8 %) using the random effects model (*I*^2^ = 68.3 %). In patients with advanced stage disease, the disease relapse rate during follow-up ranged from 0.0 to 26.7 %, with a weighted summary proportion of 8.6 % (95 % confidence interval 2.4–26.1 %) using the random effects model (*I*^2^ = 90.0 %).

**Table 4:** Results of included studies

Study (year)	Follow-up time in months (range)	No. of patients with complete remission status according to end-of-treatment FDG-PET	Disease relapse among patients in complete remission status according to end-of-treatment FDG-PET	PFS	OS	Disease relapse among patients with early stage disease in complete remission status according to end-of-treatment FDG-PET	Disease relapse among patients with advanced stage disease in complete remission status according to end-of-treatment FDG-PET
Hutchings et al. (2014) [14]	28.8^a^ (7.6–55.8)	102	7/102 (6.9 %)	2-year PFS, 93.0 %	NR	NR	NR
Picardi et al. (2014) [15]	60^a^ (4–108)	300	80/300 (26.7 %)	NR	NR	NA	80/300 (26.7 %)
Filippi et al. (2013) [16]	36^a^ (12–76)	75	1/75 (1.3 %)	NR	NR	1/75 (1.3 %)	NA
Markova et al. (2012) [17]	52^a^	59	3/59 (5.1 %)	NR	NR	NA	3/59 (5.1 %)
Okosun et al. (2012) [18]	27^a^ (12–50)	22	0/22 (0 %)	100 %	100 %	NA	0/22 (0.0 %)
Zinzani et al. (2012) [19]	45^a^ (6–100)	257	13/257 (5.1 %)	NR	NR	5/131 (3.8 %)	8/126 (6.3 %)
Barnes et al. (2011) [20]	46^a^ (6–107)	83	5/83 (6.0 %)	4-year PFS, 94 %	4-year OS, 100 %	5/83 (6.0 %)	NA
Lopci et al. (2011) [21]	25^b^ (4–60)	81	4/81 (4.9 %)	NR	NR	NR	NR
Straus et al. (2011) [22]	39.6 (4.8–60.0)	65	9/65 (13.8 %)	Estimated 2-year PFS, 89 %	NR	9/65 (13.8 %)	NA
Cerci et al. (2010) [23]	36^a^ (32–40)	93	11/93 (11.8 %)	NR	NR	NR	NR
Weighted summary disease relapse proportion			7.5 % (random effects *I* ^2^, 88.4 %)			5.7 % (random effects *I* ^2^, 68.3 %)	8.6 % (random effects *I* ^2^, 90.0 %)

^a^Median

^b^Mean

*NA* not applicable, *NR* not reported, *OS* overall survival, *PFS* progression-free survival

## Discussion

This systematic review and meta-analysis included 10 studies comprising a total number of 1137 Hodgkin lymphoma patients with complete remission status according to FDG-PET after completion of first-line therapy. Meta-analytically, approximately 7.5 % of these patients develop disease relapse during follow-up. These results were almost similar for studies that used stand-alone PET only (pooled disease relapse rate of 7.0 %). Importantly, it should be realized that more than 80 % of all Hodgkin lymphoma patients can be cured with standard first-line therapy [4]. In light of this fact, the proportion of Hodgkin lymphoma patients with a negative end-of-treatment FDG-PET scan who experience relapsed disease is relatively considerable. Therefore, end-of-treatment FDG-PET cannot confidently confirm cure and rule out residual disease. In addition, the results of the present meta-analysis show that therapeutic and prognostic studies should not only rely on end-of-treatment FDG-PET as outcome measure. Moreover, it also indicates the need for new prognostic biomarkers in Hodgkin lymphoma to improve risk stratification [3]. Of interest, another recently published meta-analysis in 727 Hodgkin lymphoma patients who had a complete remission status according to end-of-treatment FDG-PET but with a residual anatomical mass as determined by CT after first-line therapy reported these patients to have a pooled disease relapse rate of 6.8 % (95 % CI 2.6–12.5 %) during follow-up [24]. Although the included studies and patients are different between these two meta-analyses, the pooled disease relapse rates appear to be similar, with overlapping confidence intervals. These data indirectly suggest that the presence of a residual mass is not associated with a worse outcome in patients with negative end-of-treatment FDG-PET results.

Some caution is warranted when interpreting the results of this systematic review and meta-analysis. First, there was heterogeneity among the results of the included studies. Unfortunately, there were insufficient studies to use meta-regression to investigate the causes of this heterogeneity. One of the most likely explanations is that the included studies enrolled patients with varying disease stages and IPS scores, while it is known that early stage Hodgkin lymphoma has a lower risk of disease relapse than advanced-stage lymphoma has [3]. The use of different treatment regimes may also have accounted for the variability in disease relapse rates among the included studies. Second, although the overall methodological quality of included studies was reasonably good, there was a moderate risk of bias for the QUIPS domains prognostic factor and outcome measurement in about half of included studies. The former is due to the use of stand-alone PET systems, which are inferior to modern integrated PET/CT systems with regard to image quality and anatomic definition of foci of increased FDG uptake.[25]. The latter is either due to the fact that it was unclear whether disease relapses were confirmed histologically or due to the fact that histological proof of disease relapse was not available in all cases.

Classical Hodgkin lymphoma is characterized by a minority of tumor cells derived from germinal center B-cells and a vast majority of non-malignant reactive cells [26]. Despite the large number of non-malignant microenvironment cells, untreated Hodgkin lymphoma is virtually always FDG-avid [27]. High expressions of glucose transporter type 1 in Reed-Sternberg cells may play a crucial role for the relatively high FDG uptake in Hodgkin lymphoma [28]. One in vitro study investigated the effect of etoposide on cell death and on glucose uptake in a Hodgkin lymphoma cell line [29]. It was reported that decreased glucose uptake in etoposide-treated Hodgkin lymphoma cells seemed to be very closely correlated with drug-induced cell death [29]. In their experiment, apoptosis was found to occur by cumulative mechanisms, i.e., by both the direct action of etoposide and indirectly via drug-induced glucose deprivation [29]. False-negative results due to drug-mediated downregulation of glucose uptake in viable Hodgkin lymphoma cells seemed to be less of a problem in that in vitro study [29]. These previously reported data, along with the findings of the present systematic review and meta-analysis, indicate that although FDG imaging itself may intrinsically be an appropriate method to evaluate response to chemotherapy in Hodgkin lymphoma patients, FDG-PET cannot exclude (microscopic) residual disease due to its limited spatial resolution.

This systematic review and meta-analysis had some limitations. First, patients with residual or progressive disease according to end-of-treatment FDG-PET were excluded, because these patients usually undergo additional treatments, which biases outcome estimates in these patients. Therefore, the outcome of patients with a negative versus those with a positive end-of-treatment FDG-PET scan could not be meaningfully compared in this work. Nevertheless, the German Hodgkin Study Group (GHSG) HD15 trial (which included 711 patients with advanced Hodgkin lymphoma and a residual mass of >2.5 cm at end-of-treatment CT, and who also underwent end-of-treatment FDG-PET) has already shown that patients with a positive end-of-treatment FDG-PET scan have a worse outcome than those with a negative end-of-treatment FDG-PET scan, even when additional (radiation) therapy is given in the former group [30]. On the other hand, false-positive FDG-PET findings are common, and biopsy and close monitoring are required for accurate determination of residual disease [31]. Thus, both positive and negative end-of-treatment FDG-PET findings should be handled carefully before making clinical decisions. Second, the data that were provided by the individual studies that were included in this systematic review and meta-analysis did not allow for detailed subgroup analyses according to different Hodgkin lymphoma subtypes, disease stages, age, IPS scores, and applied therapy regimens, although these factors may have an impact on patient outcome. Third, in the majority of included studies, a complete remission was defined according to the Cheson 2007 response criteria [10], which consider a scan negative if FDG uptake is equal or less intense than the mediastinal blood pool activity in lesions larger than 2 cm and more than the background in lesions smaller than 2 cm. However, the new Lugano 2014 response criteria [8, 9] define complete remission status as FDG uptake equal to or lower than liver uptake with or without a residual mass at CT of any size, a method that is more reproducible [32]. As a result, the prognostic value of FDG-PET after first-line treatment may be different when the new interpretation criteria are applied. Fourth, pooled disease relapse rate among the patients with complete remission status at end-of-treatment FDG-PET was calculated based on variable follow-up times that were used in the included individual studies. In nine of 10 included studies, patients who were followed up for less than 2 years were included, whereas most relapses typically occur within the first 3 years [2–4]. Therefore, the calculated pooled disease relapse rate was probably underestimated.

In conclusion, although the disease relapse rate in Hodgkin lymphoma patients who achieve an FDG-PET-based complete remission after first-line therapy is low from an absolute point of view, it is actually high when considering the generally favorable outcome of Hodgkin lymphoma.
